# Antimicrobial susceptibility profiles and tentative epidemiological cutoff values of *Legionella pneumophila* from environmental water and soil sources in China

**DOI:** 10.3389/fmicb.2022.924709

**Published:** 2022-08-18

**Authors:** Jin-Lei Yang, Honghua Sun, Xuefu Zhou, Mo Yang, Xiao-Yong Zhan

**Affiliations:** The Seventh Affiliated Hospital, Sun Yat-sen University, Shenzhen, China

**Keywords:** *Legionella pneumophila*, antimicrobial susceptibility, epidemiological cut-off values, rifampin, clarithromycin, azithromycin, fluoroquinolones, *lpeAB*

## Abstract

Legionnaires’ disease (LD), caused by *Legionella*, including the most prevalent *Legionella pneumophila*, has been treated primarily with antibiotics. Environmental water and soil are the reservoirs for *L. pneumophila*. Studying antimicrobial susceptibility using a large number of isolates from various environmental sources and regions could provide an unbiased result. In the present study, antimicrobial susceptibility of 1464 environmental *L. pneumophila* isolates that were derived from various environmental water and soil sources of 12 cities in China to rifampin (RIF), erythromycin (ERY), clarithromycin (CLA), azithromycin (AZI), ciprofloxacin (CIP), moxifloxacin (MOX), levofloxacin (LEV), and doxycycline (DOX) was investigated, and minimum inhibitory concentration (MIC) data were obtained. We show that regarding macrolides, ERY was least active (MIC_90_ = 0.5 mg/L), while CLA was most active (MIC_90_ = 0.063 mg/L). A total of three fluoroquinolones have similar MICs on *L. pneumophila*. Among these antimicrobials, RIF was the most active agent, while DOX was the most inactive one. We observed different susceptibility profiles between serogroup 1 (sg1) and sg2-15 or between water and soil isolates from different regions. The ECOFFs were ERY and AZI (0.5 mg/L), RIF (0.002 mg/L), CIP, CLA and MOX (0.125 mg/L), LEV (0.063 mg/), and DOX (32 mg/L). Overall, two fluoroquinolone-resistant environmental isolates (0.14%) were first documented based on the wild-type MIC distribution. Not all azithromycin-resistant isolates (44/46, 95.65%) harbored the *lpeAB* efflux pump. The MICs of the ERY and CLA on the *lpeAB* + isolates were not elevated. These results suggested that the *lpeAB* efflux pump might be only responsible for AZI resistance, and undiscovered AZI-specific resistant mechanisms exist in *L. pneumophila*. Based on the big MIC data obtained in the present study, the same defense strategies, particularly against both CLA and RIF, may exist in *L. pneumophila*. The results determined in our study will guide further research on antimicrobial resistance mechanisms of *L. pneumophila* and could be used as a reference for setting clinical breakpoints and discovering antimicrobial-resistant isolates in the clinic, contributing to the antibiotic choice in the treatment of LD.

## Introduction

Legionnaires’ disease (LD), which was first identified in 1977, manifests as atypical pneumonia and can evoke severe disease and death (Fields et al., 2002). *Legionella pneumophila* (*L. pneumophila*) is the most common causative agent of LD. It is a Gram-negative, facultative intracellular opportunistic pathogen of humans and is responsible for both sporadic and epidemic community-acquired and hospital-acquired cases of LD. *Legionella pneumophila* is ubiquitously present in aquatic environments, including moist soil and water systems, where aquatic protozoa act as its natural hosts (Lau and Ashbolt, 2009). The co-evolution and everlasting pathogen–host interactions make *L. pneumophila* have the ability to infect metazoans and enable them to replicate within accidental hosts, such as lung macrophages and epithelial cells of humans, and then cause LD (Gomez-Valero and Buchrieser, 2019; Park et al., 2020). Aquatic environments, such as rivers, lakes, ponds, and garden soils, are the shelter and reservoir for *L. pneumophila* and from where it can colonize man-made environments, such as cooling towers and hot water systems, and then spread in aerosols, infecting susceptible persons (Zhan et al., 2010). Direct contact with *L. pneumophila*-contaminated water or dust is another means of infection. *L. pneumophila* is the first identified agent for LD, which accounts for approximately 91.5% of the LD cases, of which serogroup 1 (LP sg1) appears to be responsible for over 84% of diagnosed cases and 80–90% of community-acquired LD (Yu et al., 2002). Almost all human infections are due to environmental *Legionella* contamination, and only one case of probable human-to-human transmission has been reported, suggesting that LD is an environment-borne disease and *L. pneumophila*, which is hidden in the environment, is responsible for the infection (Correia et al., 2016).

Due to the overall high case fatality rate of LD (around 10%) (Chidiac et al., 2012; Beaute, 2017; Soda et al., 2017), early and active antibiotic therapy for LD patients is required. Macrolides, such as erythromycin (ERY), azithromycin (AZI), clarithromycin (CLA); fluoroquinolones (e.g., ciprofloxacin, CIP; levofloxacin, LEV; moxifloxacin, MOX); rifampicin (RIF); and tetracyclines (e.g., doxycycline, DOX) are the most commonly used antibiotics in the clinical treatment of LD (Sabria et al., 2005; Pedro-Botet and Yu, 2006; Varner et al., 2011; Levcovich et al., 2016). Macrolides have high-affinity interactions with the peptidyl transferase center (PTC) of the 50S ribosomal subunit, thereby interdicting bacterial protein synthesis (Vázquez-Laslop and Mankin, 2018; Svetlov et al., 2021). Similarly, tetracyclines inhibit protein synthesis through reversible binding to the bacterial 30S ribosomal subunit (Nguyen et al., 2014). Fluoroquinolones block bacterial DNA replication by locking DNA gyrase and topoisomerase IV (Ezelarab et al., 2018). RIF inhibits bacterial RNA polymerases, thus terminating the transcription process (Alifano et al., 2015). The four kinds of antibiotics are highly effective in treating infections caused by *L. pneumophila*, due to their intracellular permeability (Takemura et al., 2000). However, the reduced susceptibility of *L. pneumophila* to macrolides and fluoroquinolones has been reported (Massip et al., 2017; Vandewalle-Capo et al., 2017; Natas et al., 2019; Sharaby et al., 2019). Some AZI- or fluoroquinolone-resistant *L. pneumophila* isolates are found in the clinic, which is caused by a *lpeAB*-encoded efflux pump—a homolog of *Escherichia coli* AcrAB, one of the major antibiotic resistance determinants in Gram-negative bacteria—or by the mutation in the quinolone resistance-determining region (QRDR) of the *gyrA* gene of *L. pneumophila* (Bruin et al., 2014; Shadoud et al., 2015; Massip et al., 2017; Natas et al., 2019). These further indicate the urgent need to monitor the antimicrobial susceptibility of environmental isolates. The epidemiological cutoff value (ECOFF) is used to distinguish isolates with resistant mechanisms and acts as a reference in clinical treatment when an approved clinical breakpoint is not determined (Kahlmeter, 2014), which is usually the highest MIC for microorganisms lacking phenotypically detectable acquired resistance mechanisms and can be obtained from pooled MIC distributions by using the statistical goodness-of-fit tests (Turnidge et al., 2006; Karatuna et al., 2020). In the past few decades, many antimicrobial susceptibility tests (ASTs) have been conducted on *L. pneumophila* (García et al., 2000; Choi et al., 2010; Bruin et al., 2012; De Giglio et al., 2015; Xiong et al., 2016; Vandewalle-Capo et al., 2017; Koshkolda and Lück, 2018; Wilson et al., 2018; Jia et al., 2019; Natas et al., 2019; Sharaby et al., 2019; Assaidi et al., 2020; Pappa et al., 2020; Cocuzza et al., 2021); however, sufficient data to establish comprehensive ECOFFs are not currently available due to the limited number of wild-type (WT) isolates (Koshkolda and Lück, 2018). Meanwhile, a small number (dozens to hundreds) of *L. pneumophila* isolates from samples confined to a small region tested for the minimum inhibitory concentration (MIC) distribution and ECOFFs were a noteworthy issue (Al-Matawah et al., 2012). In addition, there are insufficient data on the antimicrobial susceptibility of *L. pneumophila* documented in China. Thus, knowledge of the antimicrobial susceptibility to a large number of *L. pneumophila* isolates from diverse environments in China may enable the assessment of possible antibiotic resistance of this bacterium.

In the present study, we compiled a large (∼1500 isolates) and comprehensive collection of *L. pneumophila* isolates to investigate the *in vitro* activity of RIF, ERY, CLA, AZI, CIP, MOX, LEV, and DOX against *L. pneumophila* derived from diverse environments (ponds, rivers, lakes, small streams, seawaters, puddles, fountains, potting soil, and garden soils), which could represent the most common aquatic environmental reservoirs of *L. pneumophila* in China. Antimicrobial susceptibility profiles of these isolates belonging to different serogroups (sgs) or from different environmental sources and regions were investigated. Furthermore, we aimed to determine an unbiased tentative ECOFF for these antibiotics against *L. pneumophila* since EUCAST provides widely accepted ECOFFs for these antimicrobial agents that are used for the treatment of LD.

## Materials and methods

### Ethics statement

Ethical approval was not required as only *Legionella* bacterial isolates from environmental sources were used.

### Strains, antibiotics, and culture conditions

A total of 1464 *L. pneumophila* isolates (including 329 sg1 and 1135 sg2-15 or 1079 water and 385 soil isolates) derived from 138 samples, which were obtained from 12 cities in seven provinces of China between April 2019 and January 2021, were investigated in this study. These isolates were from various aquatic sources, including water sources of rivers, sea, lakes, ponds, small streams, and grass puddles, and soil sources of the garden and potting soils (Supplementary Figure 1). The sampling and processing of water and soil samples, the isolation of *Legionella*-like bacteria, and the identification of *L. pneumophila* and *L. pneumophila* sg1 isolates were described in our previous study (Zhan et al., 2022). The *L. pneumophila* strain ATCC 33152 was used as a reference strain, according to EUCAST guidelines. A total of eight antibiotics were tested: RIF, ERY, CLA, AZI, CIP, MOX (Rhawn, Shanghai, China), LEV (Solarbio, Beijing, China), and DOX (Macklin, Shanghai, China). The testing concentration range is 0.016–32.0 mg/L for ERY and DOX; 0.004–8.0 mg/L for CIP, CLA, AZI, MOX, and LEV; and 0.0000625–0.125 mg/L for RIF based on the data obtained from the reference strain ATCC33152. The *Legionella* bacteria were inoculated on buffered charcoal yeast extract agar supplemented with 1g/L α-ketoglutarate, 0.25 g/L L-cysteine, and 0.4 g/L ferric pyrophosphates (BCYEα) and then incubated at 37 °C with 5% v/v CO2 atmosphere with a higher humidity for 72-96 h before the antibiotic susceptibility test (AST).

### Minimum inhibitory concentration determination

Minimum inhibitory concentrations (MICs) were determined by using the broth microdilution (BMD) test using buffered yeast extract broth supplemented with 1g/L α-ketoglutarate, 0.25 g/L L-cysteine, and 0.4 g/L ferric pyrophosphates (BYE) on 96-well microtiter plates as it can provide unbiased MICs due to the absence of charcoal and it was the internationally agreed gold standard for *Legionella* species (Portal et al., 2021b). In brief, a single colony of *Legionella* was isolated from a BCYEα plate and inoculated in a BYE broth, adjusting the optical density of the bacterial suspension to OD600 = 0.1 (∼1.0 × 10^8^/mL) in a 1.5-mL tube. A final *Legionella* bacterial concentration of ∼10^5^/mL in each well was obtained by dilution. A volume of 20 μL of each antibiotic solution was then added to microtiter wells in 2-fold decreasing concentrations. The final volume of each well was 100 μL, containing 10^4^ *Legionella* bacteria. A blank well consisting of 100 μL of the BYE broth was used as a negative control. The plates were sealed and incubated for 48 h without agitation at 37^°^C before determining the MIC by observing the presence or absence of turbidity. The MIC was defined as the lowest concentration of antibiotics yielding complete inhibition of visible growth. Each isolate was tested at least two times to verify the results.

### MIC_50_/MIC_90_, epidemiological cutoff value, and detection of antibiotic resistance isolates

The MICs that inhibited the growth of 50% and 90% of strains were defined as MIC_50_ and MIC_90_, respectively. The ECOFF is used to separate bacterial populations based on MIC distributions and is defined as the highest MIC value of isolates that are not known to have resistance (Chidiac et al., 2012). The ECOFFinder program (version 2010-v21), which was raised by the EUCAST, was applied to fit a log-normal distribution curve for the number of isolates that had MICs for *L. pneumophila*. Theoretically, no less than 95% of WT isolates should be encompassed in the ECOFF; thus, the 97.5% endpoints of value were defined as the ECOFF of a specific antibiotic against *L. pneumophila*, expressed as WT ≤ X mg/L. We defined isolates with an MIC higher than ECOFF are resistant isolates, whereas others are susceptible ones.

### Determination of possible resistance mechanism

More and more antibiotic-resistant isolates are found in clinical *L. pneumophila* isolates (Bruin et al., 2014; Natas et al., 2019; Cocuzza et al., 2021). These isolates are mainly AZI-resistant, and resistant isolates against other antibiotics are rare (Bruin et al., 2014; Jia et al., 2019). *L. pneumophila* is a typical environment-borne pathogen. Investigation of antibiotic susceptibility profiles of *L. pneumophila* from various environmental sources is crucial in generating antibiotic treatment recommendations for clinical infection. Because macrolides, including AZI, are the first-line antimicrobial agents for the treatment of LD, we focus on the molecular mechanism of AZI-resistant isolates as they are shown to have a high frequency in *L. pneumophila* (Jia et al., 2019; Natas et al., 2019; Cocuzza et al., 2021). Many macrolide resistance-mediating mechanisms were found in bacteria, including enzymatic modifications and 23S rRNA gene mutation, as well as ribosomal accessory protein-coding genes including *rplD* (protein L4) and *rplV* (protein L22) (Prats-van der Ham et al., 2018; Abushaheen, 2020). However, an efflux pump encoded by *lpeA and lpeB* (designated as *lpeAB*) is mainly the cause of AZI resistance in *L. pneumophila* (Massip et al., 2017; Jia et al., 2019; Natas et al., 2019). Therefore, we designed a multiplex PCR method to detect the presence of the *lpeAB* efflux pump in the environmental isolates with AZI resistance. A total of 123 reference *L. pneumophila* strains that harbored *lpeAB* efflux were utilized for an efficient primer design. The detailed information of these strains including names, genomic sequences, GenBank accession numbers, and *lpeA/lpeB* gene locations in the sequences is shown in Supplementary Table 1. Overall, six alleles were found both on *lpeA* and *lpeB* genes. Based on the conservative sequence of *lpeAB* genes, two pairs of primers were designed as follows: lpeA_F: CTGTWGTAAGTATTTACGACCC (W indicates degenerate bases and is equal to A and T), lpeA_R: GGTGTCTTCGTCGAGCA; lpeB_F: CA TCCTGTAATCACCATCATC, lpeB_R: CAACGGAAGCA ACACCTTG. These primers could not only detect the presence of *lpeAB* but also define if the two genes were correctly arranged in spatial.

### Statistical analysis

Continuous variables (log MICs) were compared using Student’s *t*-test or one-way ANOVA. For categorical variables, we calculated the frequencies and percentages of isolates in each category by chi-square or Fisher’s exact tests. Non-parametric Spearman’s correlation analysis was used to analyze the relationship between different antibiotics against *L. pneumophila* isolates. Statistical analyses were performed using SPSS 25.0 (IBM software). A principal component analysis (PCA) for MICs of the eight antibiotics was performed using Origin 2021b (OriginLab). *P* < 0.05 was considered statistically significant. Simpson’s diversity index was used to measure the homogeneity of antibiotic susceptibility of the isolates using the formula: D = 1-Σ(Ni/N)^2^.

## Results and discussion

### Minimum inhibitory concentration ranges and MIC_50_/MIC_90_ for *Legionella pneumophila* isolates from environmental sources

**FIGURE 1:**
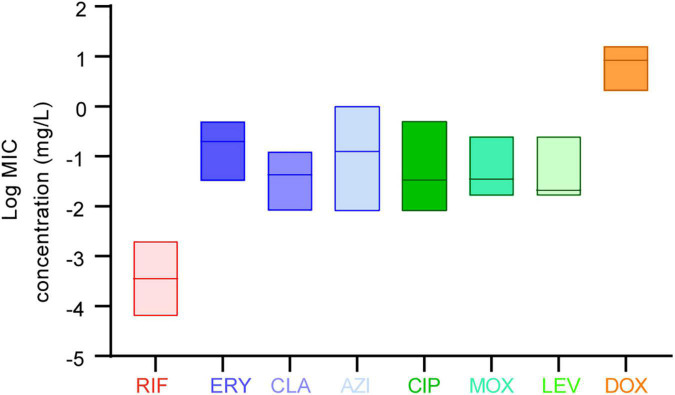
Average minimum inhibitory concentrations (MICs) among the eight antibiotics against *L. pneumophila.* Data are shown as floating bars with the max, min, and mean values.

The *in vitro* susceptibility profiles of the 1464 *L. pneumophila* isolates against the eight antibiotics, as judged by MIC distributions, are shown in Table 1. From the overall MIC distribution of the 1464 *L. pneumophila* isolates, almost all tested ones were inhibited by low concentrations of RIF, fluoroquinolones, and macrolides. ERY yielded MICs ranging between 0.031 and 0.5 mg/L. Similar results were found in CIP (0.008–0.5 mg/L), which had the same upper limit but a lower limit as compared with ERY. By contrast, AZI produced a higher upper limit of MIC than CIP (0.008–1 mg/L). This result was following the average MICs of each antibiotic against *L. pneumophila* (Figure 1). One dilution lower MIC range of MOX and LEV was observed (0.016–0.25 mg/L) as compared with ERY. RIF was most active, with the highest MIC being 0.002 mg/L, whereas DOX was most inactive, with the lowest MIC being 2 mg/L. Based on this result, DOX seems to be not recommended to treat LD, although it is the second choice for patients with minor pneumonia (Torre et al., 2018). We did not find significant MIC range differences between sg1 and sg2-15 isolates. Regarding the control strain, *L. pneumophila* sg 1 ATCC 33152 (two repetitions) showed higher sensitivities (MIC of ATCC33152 ≤ MIC_50_ of the environmental isolates) to all the eight antibiotics, including RIF (0.0005 mg/L), ERY (0.125 mg/L), CLA (0.016 mg/L), AZI (0.25 mg/L), CIP (0.063 mg/L), MOX (0.031 mg/L), LEV (0.031 mg/L), and DOX (8 mg/L). MIC diversity among the isolates was highest in ERY, while was lowest in MOX (0.70 vs. 0.23), indicating more mechanisms might be existed in confronting ERY for *L. pneumophila*, and vice versa. Previous studies using the BMD method to perform susceptibility testing showed variability in the MIC_50_, MIC_90_, and MIC ranges (García et al., 2000; Choi et al., 2010; Xiong et al., 2016; Koshkolda and Lück, 2018; Wilson et al., 2018; Assaidi et al., 2020; Cocuzza et al., 2021) (Table 2). Our data are comparable to some of these results (Table 2). The variability may be because of the limited number of isolates used in those studies and the influence of regional differences for the *L. pneumophila* isolates, raising the importance of using numerous isolates from different sources and using the standard protocol to define ECOFFs for *L. pneumophila*. Given that LD is a typical environment-borne disease and all the strains that caused infections are from environments, our results obtained based on numerous environmental isolates could indicate the antimicrobial susceptibility to the clinical isolates.

**TABLE 1:** Minimum inhibitory concentration (MIC) data of eight antimicrobials for the 1464 *L. pneumophila* isolates.

Antibiotics	No. of *L.pneumophila* isolates inhibited at indicated concentrations (mg/L)																								
		0.0000625	0.000125	0.00025	0.0005	0.001	0.002	0.004	0.008	0.016	0.031	0.063	0.125	0.25	0.5	1	2	4	8	16	32	MIC_50_	MIC_90_	MIC range	MIC diversities
RIF	All	18	77	580	749	37	3															0.0005	0.0005	0.0000625–0.002	0.58
	Sg1	1	20	183	119	5	1															0.00025	0.0005	0.0000625–0.002	0.56
	Sg2-15	17	57	397	630	32	2															0.0005	0.0005	0.0000625–0.002	0.57
ERY	All										3	117	552	494	298							0.25	0.5	0.031–0.5	0.70
	Sg1											63	182	74	10							0.125	0.25	0.063–0.5	0.61
	Sg2-15										3	54	370	420	288							0.25	0.5	0.031–0.5	0.69
CLA	All							1	3	46	748	661	5									0.031	0.063	0.004–0.125	0.53
	Sg1									13	233	82	1									0.031	0.063	0.008–0.125	0.44
	Sg2-15							1	3	33	515	579	4									0.063	0.063	0.004–0.125	0.53
AZI	All								1	0	2	264	1005	146	33	13						0.125	0.25	0.008–1	0.49
	Sg1											92	196	11	30							0.125	0.25	0.063–0.5	0.56
	Sg2-15								1	0	2	172	809	135	3	13						0.125	0.25	0.008–1	0.46
CIP	All								3	103	1160	195	1	0	2							0.031	0.063	0.008–0.5	0.35
	Sg1								1	12	276	37	1	0	2							0.031	0.063	0.008–0.5	0.28
	Sg2-15								2	91	884	158										0.031	0.063	0.008–0.063	0.37
MOX	All									20	1327	51	64	2								0.031	0.031	0.016–0.25	0.18
	Sg1									3	307	15	2	2								0.031	0.031	0.016–0.25	0.13
	Sg2-15									17	1020	36	62									0.031	0.031	0.016–0.125	0.19
LEV	All									965	451	46	0	2								0.016	0.031	0.016–0.25	0.47
	Sg1									182	140	5	0	2								0.016	0.031	0.016–0.25	0.51
	Sg2-15									783	311	1										0.016	0.031	0.016–0.063	0.41
DOX	All																1	58	1279	126		8	8	2–16	0.23
	Sg1																	19	285	25		8	8	4–16	0.24
	Sg2-15																1	39	994	101		8	8	2–16	0.22

The first column of the tables shows names of the antibiotics. The antibiotics belonging to the same class are filled with same color, shown as light red for rifampicin, light blue for macrolides, light green for fluoroquinolones, and light orange for tetracyclines. Other cells filled with colors indicate the concentration ranges of the antibiotics that were used for MIC determination.

**TABLE 2:** Susceptibility of antimicrobials against *Legionella pneumophila* by the BMD method described by other articles.

Antibiotics	MIC_50_ (mg/L)	MIC_90 (_ mg/L)	MIC range (mg/L)	Number of isolates	Sg of isolates	Sources	Regions of isolates	References
RIF	0.0005	0.0005	0.00012–0.001	109	Sg1	Clin.	France	Vandewalle-Capo et al., 2017
	≤ 0.008	≤0.008	≤ 0.008–0.15	58	Undefined	Env.	Northern Italy	Cocuzza et al., 2021
	≤ 0.008	≤0.008	≤ 0.008	24	Sg1	Env.	Northern Italy	Cocuzza et al., 2021
	≤ 0.008	≤0.008	≤ 0.008–0.15	34	Sg2-15	Env.	Northern Italy	Cocuzza et al., 2021
	0.001	0.001	0.001	92	Undefined	Clin.	England and Wales	Wilson et al., 2018
	0.032	0.094	0.016–0.25	20	Sg1	Env.	Morocco	Assaidi et al., 2020
	0.032	0.064	0.016–0.19	38	Sg2-15	Env.	Morocco	Assaidi et al., 2020
	0.0005	0.0005	0.0000625–0.002	1464	Undefined	Env.	China	This study
ERY	0.125	0.5	0.03–1	109	Sg1	Clin.	France	Vandewalle-Capo et al., 2017
	0.06	0.12	0.015–8	58	Undefined	Env.	Northern Italy	Cocuzza et al., 2021
	0.12	0.5	0.015–0.5	24	Sg1	Env.	Northern Italy	Cocuzza et al., 2021
	0.06	0.12	0.015–8	34	Sg2-15	Env.	Northern Italy	Cocuzza et al., 2021
	0.25	0.5	0.06–1	92	Undefined	Clin.	England and Wales	Wilson et al., 2018
	0.25	0.75	0.19–2	20	Sg1	Env.	Morocco	Assaidi et al., 2020
	0.19	0.75	0.064–0.75	38	Sg2-15	Env.	Morocco	Assaidi et al., 2020
	0.06	0.12	0.03–1	270	Undefined	Clin. + Env.	Spain	García et al., 2000 *
	0.125	0.5	N/A	40	Undefined	Env.	China	Xiong et al., 2016
	0.125	0.25	N/A	12	Sg1	Env.	China	Xiong et al., 2016
	0.25	0.5	N/A	28	Sg2-14	Env.	China	Xiong et al., 2016
	0.25	0.5	0.031–0.5	1464	Undefined	Env.	China	This study
CLA	0.032	0.032	0.004–0.064	109	Sg1	Clin.	France	Vandewalle-Capo et al., 2017
	0.094	0.25	0.032–1	20	Sg1	Env.	Morocco	Assaidi et al., 2020
	0.064	0.38	0.032–0.75	38	Sg2-15	Env.	Morocco	Assaidi et al., 2020
	0.007	0.015	0.0004–0.03	270	Undefined	Clin. + Env.	Spain	García et al., 2000 *
	0.008	0.031	0.004–0.031	27	Undefined	Env.	South Korea	Choi et al., 2010
	0.12	0.25	0.004–0.5	15	Sg1	Clin. + Env.	South Korea	Choi et al., 2010
	0.016	0.5	0.004–0.5	16	Sg2-15	Clin. + Env.	South Korea	Choi et al., 2010
	0.031	0.063	0.004–0.125	1464	Undefined	Env.	China	This study
AZI	0.06	0.5	0.015–2	109	Sg1	Clin.	France	Vandewalle-Capo et al., 2017
	0.03	0.12	≤ 0.008-8	58	Undefined	Env.	Northern Italy	Cocuzza et al., 2021
	0.06	0.5	0.015–1	24	Sg1	Env.	Northern Italy	Cocuzza et al., 2021
	0.015	0.03	≤ 0.008-8	34	Sg2-15	Env.	Northern Italy	Cocuzza et al., 2021
	0.125	0.38	0.064–1.5	20	Sg1	Env.	Morocco	Assaidi et al., 2020
	0.19	0.5	0.047–0.75	38	Sg2-15	Env.	Morocco	Assaidi et al., 2020
	0.062	0.25	N/A	40	Undefined	Env.	China	Xiong et al., 2016
	0.062	0.25	N/A	12	Sg1	Env.	China	Xiong et al., 2016
	0.062	0.5	N/A	28	Sg2-14	Env.	China	Xiong et al., 2016
	0.016	0.062	0.004–0.062	27	Undefined	Env.	South Korea	Choi et al., 2010
	0.031	0.062	0.004–0.062	16	Sg1	Clin. + Env.	South Korea	Choi et al., 2010
	0.016	0.062	0.004–0.062	15	Sg2-15	Clin. + Env.	South Korea	Choi et al., 2010
	0.125	0.25	0.008–1	1464	Undefined	Env.	China	This study
CIP	0.016	0.032	0.008–0.064	109	Sg1	Clin.	France	Vandewalle-Capo et al., 2017
	0.015	0.06	≤ 0.008–0.5	58	Undefined	Env.	Northern Italy	Cocuzza et al., 2021
	0.015	0.03	≤ 0.008–0.03	24	Sg1	Env.	Northern Italy	Cocuzza et al., 2021
	0.015	0.06	≤ 0.008–0.5	34	Sg2-15	Env.	Northern Italy	Cocuzza et al., 2021
	0.015	0.015	0.004–0.25	92	Undefined	Clin.	England and Wales	Wilson et al., 2018
	0.125	0.75	0.064–1.5	20	Sg1	Env.	Morocco	Assaidi et al., 2020
	0.19	0.5	0.047–0.75	38	Sg2-15	Env.	Morocco	Assaidi et al., 2020
	0.015	0.03	0.0018–0.03	270	Undefined	Clin. + Env.	Spain	García et al., 2000 *
	0.031	0.031	0.004–0.062	40	Undefined	Env.	China	Xiong et al., 2016
	0.031	0.031	N/A	12	Sg1	Env.	China	Xiong et al., 2016
	0.031	0.031	N/A	28	Sg2-14	Env.	China	Xiong et al., 2016
	0.016	0.031	0.004–0.12	27	Undefined	Env.	South Korea	Choi et al., 2010
	0.031	0.25	0.008–0.5	16	Sg1	Clin. + Env.	South Korea	Choi et al., 2010
	0.016	0.5	0.004–0.5	15	Sg2-15	Clin. + Env.	South Korea	Choi et al., 2010
	0.031	0.063	0.008–0.5	1464	Undefined	Env.	China	This study
MOX	0.032	0.032	0.008–0.064	109	Sg1	Clin.	France	Vandewalle-Capo et al., 2017
	0.125	0.125	0.03–0.25	92	Undefined	Clin.	England and Wales	Wilson et al., 2018
	0.25	0.5	0.19–3	20	Sg1	Env.	Morocco	Assaidi et al., 2020
	0.25	0.5	0.064–1.5	38	Sg2-15	Env.	Morocco	Assaidi et al., 2020
	0.031	0.062	0.004–0.125	40	Undefined	Env.	China	Xiong et al., 2016
	0.031	0.062	N/A	12	Sg1	Env.	China	Xiong et al., 2016
	0.031	0.062	N/A	28	Sg2-14	Env.	China	Xiong et al., 2016
	0.031	0.031	0.016–0.25	1464	Undefined	Env.	China	This study
	0.016	0.032	0.004–0.032	109	Sg1	Clin.	France	Vandewalle-Capo et al., 2017
LEV	0.015	0.03	≤ 0.008–0.5	58	Undefined	Env.	Northern Italy	Cocuzza et al., 2021
	0.015	0.03	≤ 0.008–0.03	24	Sg1	Env.	Northern Italy	Cocuzza et al., 2021
	≤ 0.008	0.015	≤ 0.008–0.5	34	Sg2-15	Env.	Northern Italy	Cocuzza et al., 2021
	0.06	0.125	0.03–0.25	92	Undefined	Clin.	England and Wales	Wilson et al., 2018
	0.064	0.25	0.064–1	20	Sg1	Env.	Morocco	Assaidi et al., 2020
	0.064	0.38	0.047–1	38	Sg2-15	Env.	Morocco	Assaidi et al., 2020
	0.007	0.015	0.007–0.03	270	Undefined	Clin. + Env.	Spain	García et al., 2000 *
	0.016	0.031	0.004–0.062	40	Undefined	Env.	China	Xiong et al., 2016
	0.031	0.031	N/A	12	Sg1	Env.	China	Xiong et al., 2016
	0.016	0.031	N/A	28	Sg2-14	Env.	China	Xiong et al., 2016
	0.016	0.031	0.016–0.25	1464	Undefined	Env.	China	This study
	1	2	0.12–2	109	Sg1	Clin.	France	Vandewalle-Capo et al., 2017
DOX	2	4	0.19–8	20	Sg1	Env.	Morocco	Assaidi et al., 2020
	1.5	4	0.19–8	38	Sg2-15	Env.	Morocco	Assaidi et al., 2020
	8	16	0.5–16	40	Undefined	Env.	China	Xiong et al., 2016
	8	16	N/A	12	Sg1	Env.	China	Xiong et al., 2016
	8	16	N/A	28	Sg2-14	Env.	China	Xiong et al., 2016
	8	8	2–16	1464	Undefined	Env.	China	This study

Env. indicates environmental sources; Clin. indicates clinical source.

Cells filled in gray indicate similar results to those obtained by the present study in MIC_50_, MIC_90_, and MIC range (filled in light brown, defined as no more than one dilution difference in the parameter, and two of three parameters match are defined as similar).

*Study enrolled 271 strains (270 L. pneumophila and 1 L. longbeachae) for AST; thus, the results could only roughly represent the susceptibility of antimicrobials against L. pneumophila. N/A indicates not available.

### Distinct antimicrobial susceptibility profiles of *Legionella pneumophila* isolates with different serogroups, environmental origins, or regions

**FIGURE 2:**
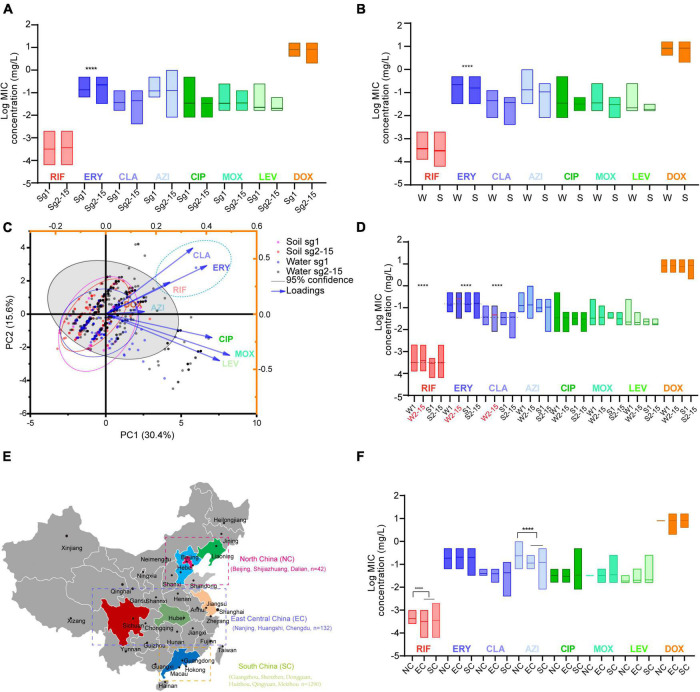
Antimicrobial susceptibility profiles of *L. pneumophila* belonging to different sgs or from different environmental sources or regions in China. **(A)** Antibiotic susceptibility profiles of isolates belonging to different sgs. **(B)** Antibiotic susceptibility profiles of isolates from different sources. W, water sources; S, soil sources. **(C)** PCA biplot of MICs of the isolates. Isolates are shown as dots and colored by groups based on the sources and sgs. Indices are given as lines with arrows and colored. The configuration of indices in the biplot represents the relationship between variables and principal components. The gray shadow indicates 95% confidence for sg2-15 isolates from water sources. A dashed ellipse indicates significantly correlated variables. **(D)** Antimicrobial susceptibility profiles of isolates belonging to different sgs and from different sources. W1, sg1 isolates from water sources; W2-15, sg2-15 isolates from water sources; S1, isolates from soil sources; S2-15, sg2-15 isolates from water sources. Data are shown as floating bars with the max and min and mean values. Dotted lines indicate that log MICs are different in W2-15 isolates when compared with the other three types of isolates. **(E)** Regions of China where the tested isolates were obtained are shown. Cities where the isolates were obtained in each region are shown. **(F)** Antibiotic susceptibility profiles of isolates from different regions of China. Colored regions indicate the provinces where the isolates are from. We defined those MICs were 1.25 times higher/lower (log MIC gap > 0.969) than the contrast with *P* < 0.05 as significant. ****P < 0.0001.

Average MICs obtained by antibiotics against sg2-15 isolates were significantly higher in ERY than those against the sg1 isolates (Figure 2A). We could also observe that MICs obtained by ERY against the isolates derived from China’s water sources were higher than those from soil sources (Figure 2B). PCA indicated that sg2-15 isolates from water samples displayed a specific nature when compared to sg2-15 isolates from soil or sg1 from both soil and water, of which CLA, ERY, and RIF might have a homologous contribution to the nature (Figure 2C). Further study suggested that sg2-15 isolates from water showed significantly decreased susceptibility to RIF, ERY, and CLA (Figure 2D). These results together indicated that antimicrobial susceptibility profiles of *L. pneumophila* belonging to different sgs or from different environmental sources were distinct. Many studies demonstrated antimicrobial susceptibility in a limited number of *L. pneumophila* isolates from small geographic areas (Xiong et al., 2016; Graells et al., 2018; Koshkolda and Lück, 2018; Torre et al., 2018; Wilson et al., 2018; Cocuzza et al., 2021). As China is a country with a huge land area, antimicrobial susceptibility in *L. pneumophila* from different geographic may be distinct. Therefore, MICs of the eight antibiotics against isolates from northern, central, eastern, and southern China were compared (Figure 2E). We found that average MICs of RIF and AZI against northern isolates were significantly higher than those against isolates from other regions of China (Figure 2F), indicating decreased susceptibility of these antibiotics to those isolates from northern China. Antibiotics enter the environment *via* waste produced by humans, agricultural food and animal production, pharmaceutical production, etc. This process may lead to a susceptibility decrease by natural selection. Thus, the RIF and AZI susceptibility decrease might be caused by relatively high environmental concentrations of the two antibiotics in north China, although some articles reported that the Pearl River basin, in which many south cities are located, has the highest antibiotics emission densities (Qiao et al., 2018).

### Principal component analysis and correlation networks for minimum inhibitory concentrations revealed possible shared strategies within *Legionella pneumophila* against antibiotics

**FIGURE 3:**
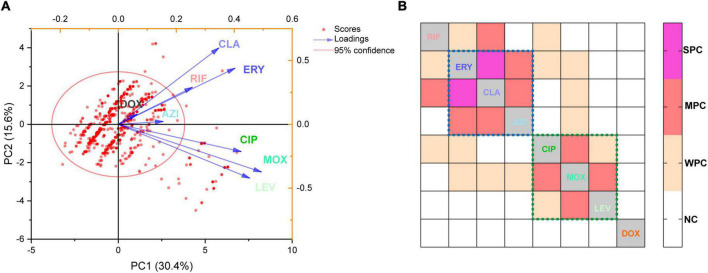
Principal component analysis and correlation matrices for the minimum inhibitory concentrations (MICs) were obtained from 1464 *L. pneumophila* isolates. **(A)** PCA of indices of the isolates. Isolates are shown as dots and colored red. Indices are shown as lines with arrows and colored. The configuration of indices in the plot represented the relationship between variables and principal components. **(B)** Correlation matrix for isolates’ indices (MICs). WPC, weak positive correlation, 0.2 < *r* ≤ 0.3; MPC, moderate positive correlation, 0.3 < *r* ≤ 0.5; SPC, strong positive correlation, *r* > 0.5. NC, no correlation. The largest r was found between CLA and ERY (*r* = 0.51). The dotted lines indicate that these antibiotics (in the dotted lines with the same color) belong to the same class.

A biplot *via* PCA indicated the configuration of MIC distributions of the eight antibiotics on the 1464 *L. pneumophila* isolates in China, as shown in Figure 3A. Loading signs of the antibiotic MICs indicates whether they are positively or negatively correlated. We found that the susceptibility of antibiotics belonging to the same class (e.g., macrolides and fluoroquinolones) was positively correlated, for they have signed with the same directions. This is in line with our expectations because antibiotics belonging to the same class always have their particular mechanism to kill microorganisms, and a confrontation mechanism has also been developed. To our surprise, RIF, which had a distinct direct antimicrobial mechanism, may have a positive correlation with macrolides (Figure 3A). Correlation matrix analysis revealed that MICs of antibiotics belonging to the same class were positively correlated (e.g., among ERY, CLA, and AZI, or among MOX, LEV, and CIP), indicating the most similar mechanism against *L. pneumophila* by the same class of antibiotics or confrontation mechanism against them by *L. pneumophila* (Figure 3B). MICs of RIF to *L. pneumophila* were positively correlated with CLA, ERY, and CIP, with CLA having a moderate correlation (r = 0.32, P < 0.0001, Figure 3B). This also holds for isolates belonging to different sgs or from different environmental sources, especially for sg1 and water isolates (Supplementary Figure 2). RIF plus CLA was a standard of care for many infections, including *Mycobacterium ulcerans* and *Mycobacterium avium* infections (Akiyama et al., 2019; Almeida et al., 2019). However, the interaction of RIF and CLA to the cytochrome P450 may influence the therapy (Akiyama et al., 2019). Thus, clinical use of the RIF-CLA combination to treat LD may not benefit not only because of unsatisfactory clinical outcomes that have been observed in other intracellular bacteria, such as *Mycobacteria* (Akiyama et al., 2019), and in *L. pneumophila* pneumonia patients who had a longer length of stay and a trend toward higher bilirubin levels when using CLA plus RIF (Grau et al., 2006), but also because of the probability of reduced susceptibility against *L. pneumophila*. The *RpoB* gene mutation in the RIF resistance-determining region (RRDR) is the major cause of decreased RIF susceptibility in many bacteria (Campbell et al., 2001). In addition, a few putative efflux pumps (*viz.*, Rv1258c, Rv1410c, Rv1819c, and PstB) have been observed to play a role in regulating RIF susceptibility in another intracellular bacterium *Mycobacterium tuberculosis* (Pang et al., 2013; Narang et al., 2019). Efflux pumps that used two or more antibiotics as substrates were observed in bacteria, including the RND efflux pumps (fluoroquinolones, macrolides, and aminoglycosides), the MFS efflux pumps (isoniazid, fluoroquinolones, rifampicin, tetracyclines, etc.), and the ABC efflux pumps (macrolides, streptogramins, and fluoroquinolones) (Thakur et al., 2021). We speculated that special defense strategies against both RIF and CLA may exist in *L. pneumophila*, probably due to undiscovered efflux pumps used as substrates or similar infiltration mechanism of the two antibiotics in *L. pneumophila*, which deserve further research. Although DOX showed less efficiency in inhibiting *L. pneumophila in vitro* (Figure 1 and Table 1), the uncorrelated susceptibility to all other antimicrobials implied it was a potential supplement in confronting resistant strains of other antibiotics (Figure 3B).

### Epidemiological cutoff value and the presence of antibiotic resistance isolates

**FIGURE 4:**
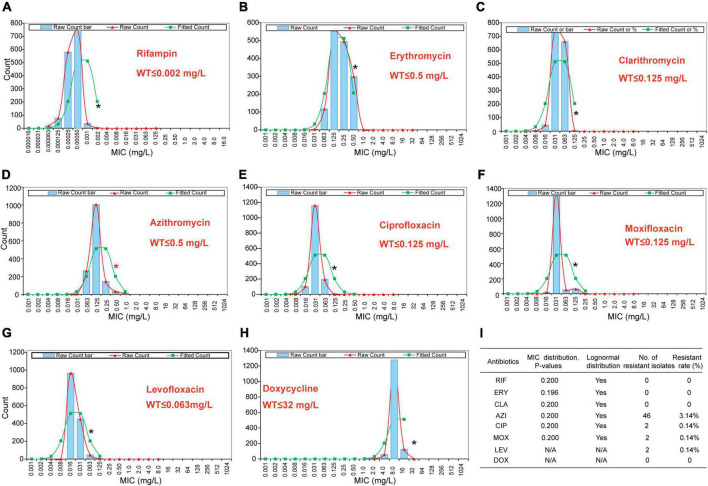
Wild-type cutoff (COWT) of the eight antibiotics against *L. pneumophila* using the ECOFFinder method. **(A–H)** MIC distributions of the eight antibiotics, fitted curves, and ECOFF were obtained by the ECOFFinder. **(I)** Relevant data table obtained by the ECOFFinder. Asterisks indicate the possible largest WT MICs.

The primary MIC distribution of the eight antimicrobials against *L. pneumophila* is shown in Figures 4A–H. Raw and fitted curves were obtained by ECOFFinder. Due to the limited number of MICs for LEV and DOX (only four for each), the normal distribution could not be calculated for the two antibiotics (Figure 4I), they still showed good shape distributions as the other six antibiotics. As a result, the ECOFF was defined as 0.002 mg/L for RIF; 0.5 mg/L for ERY; 0.125 mg/L for CIP, CLA, and MOX; 0.5 g/L for AZI; 0.063 mg/L for LEV; and 32 mg/L DOX (Figures 4A–H). These results were comparable to those obtained by using BMD methods but may be more accurate due to a large number of isolates (Table 3). Jia et al. (2019) defined ECOFFs based on 149 strains using the E-test method and obtained a 2-fold higher value for AZI (1 mg/L) and ERY (1 mg/L); 8-fold higher value for MOX (1 mg/L) and LEV (0.5 mg/L); and 16-fold higher values for the and RIF (0.031 mg/L) when compared with our data. Bruin et al. established the ECOFFs of 10 antimicrobials for sg1 using 183 clinical isolates by the E-test (Bruin et al., 2012). Sharaby et al. (2019) established the ECOFFs of 10 antimicrobials for *L. pneumophila* using 93 environmental and 12 clinical isolates by using the E-test (Sharaby et al., 2019). They showed 2- to16-fold higher MICs in most of the antibiotics, which were similar to Jia et al.’s report. Conversely, their E-test (E-test methods as indicated in Table 3) for DOX in *L. pneumophila* sg1 gained about 1/80∼1/2-fold MICs and 1/64∼1/4 ECOFFs when compared with our data and Portal et al.’s data (Tables 1, 3) (Bruin et al., 2012; Portal et al., 2021b). Given that charcoal in the BCYEα plate biases the MICs for many antibiotics, including RIF, macrolides, and tetracycline (Bornstein et al., 1985), two or more folds of high MICs or ECOFFs in most of the antimicrobials could be obtained when compared with the BMD method (Portal et al., 2021b); however, this seemed not to hold for DOX. Thus, a standardized method for *Legionella* is urgent to ensure that the MIC data remain consistent (Portal et al., 2021a). Portal et al. (2021b) obtained an ECOFF for AZI based on the BMD methods (Portal et al., 2021b). Natas et al. (2019); Portal et al. (2021b) defined MICs of AZI based on a bimodal MIC distribution of 122 isolates. They all obtained an ECOFF WT ≤ 0.25 mg/L for AZI. Natas et al. (2019) also reported that isolates with MICs ≥ 0.5 mg/L all harbored resistant genes (*lpeAB*) and could be defined as resistant isolates. These results were roughly consistent with our results obtained by the ECOFFinder, although we did not find an obvious bimodal distribution of MIC on the 1464 isolates. Vandewalle-Capo et al. (2017) reported the ECOFF WT ≤ 2 mg/L for AZI by using the BYD method. However, owing to the seemly trimodal of MIC distribution, a WT ≤ 0.25 mg/L might be the “real” ECOFF (Vandewalle-Capo et al., 2017), which was also roughly consistent with our results. For consistency, we also tentatively defined those isolates with MIC ≥ 0.5 mg/L for azithromycin as resistant ones. Therefore, based on the WT ECOFFs of the present study and AZI-resistant thresholds reported by other studies, some resistant isolates to all the fluoroquinolones (ciprofloxacin, moxifloxacin, and levofloxacin) (two isolates, 0.14%) and azithromycin (46 isolates, 3.14%) were found (Figure 4I). Few studies have reported fluoroquinolone-resistant *L. pneumophila* (Bruin et al., 2014). Bruin et al. (2014); Shadoud et al. (2015) reported the isolation of ciprofloxacin-resistant *L. pneumophila* sg1 isolates (MIC = 2 mg/L, E-test method) from a pneumonia patient, probably due to the *gyrA* mutation during antimicrobial therapy. Fluoroquinolone-resistant strains from environmental sources were not documented yet, and our findings revealed the hidden isolates in the environments and emphasized the need for environmental surveillance and susceptibility testing of *L. pneumophila* isolates. Given that the two isolates were resistant to all fluoroquinolones (CIP, MOX, and LEV) based on the WT distribution of MIC, resistance-associated mutations in the QRDR of DNA gyrase and topoisomerase IV coding genes may exist and requires further research. The two studies that utilized limited numbers (122 and 149 strains) of *L. pneumophila* composed of environmental and clinical strains from Norway or China showed that frequencies of AZI-resistant ones were both about 17% (21/122 and 25/149), and environmental strains displayed similar frequencies of resistant ones with the clinical strains (Jia et al., 2019; Natas et al., 2019). In a study with 58 environmental isolates from northern Italy, 6.9% of AZI-resistant *L. pneumophila* were found with an MIC ≥ 0.5 mg/L (BMD method) (Cocuzza et al., 2021). In addition, Vandewalle-Capo et al. demonstrated that 23.85% (26/109) of L. pneumophila clinical sg1 strains had MICs ≥ 0.5 mg/L, and 11.01% (12/109) of which were AZI-resistant ones based on the ECOFF WT ≤ 2 mg/L they obtained (BMD method) (Vandewalle-Capo et al., 2017). However, our big data indicated that the frequency of AZI-resistant *L. pneumophila* in the environmental reservoir might be less (3.14%) than ever thought.

**TABLE 3:** Epidemiological cutoff values (ECOFFs) of antimicrobials for *L. pneumophila* that are described in other articles.

Antibiotics	ECOFFs (WT ≤ X mg/L)	Methods	Number of isolates	Sg of isolates	Sources	Regions of isolates	Ref.
	0.001	BMD	109	Sg1	Clin.	France	Vandewalle-Capo et al., 2017
	0.008	BMD	50	Undefined	Clin. + Env.	England and Wales	Portal et al., 2021b *
RIF	0.008	BMD	92	Undefined	Clin.	England and Wales	Wilson et al., 2018 *
	0.032	E-test	183	Sg1	Clin.	Netherlands	Bruin et al., 2012
	0.032	E-test	100	Undefined	Env.	Southern Italy	De Giglio et al., 2015 *
	0.032	E-test	122	Undefined	Clin. + Env.	Norway	Natas et al., 2019 *
	0.032	E-test	149	Sg1	Clin. + Env.	China	Jia et al., 2019
	0.063	E-test	105	Undefined	Clin. + Env.	Northern Israel	Sharaby et al., 2019
	0.002	BMD	1464	Undefined	Env.	China	This study
ERY	1	BMD	109	Sg1	Clin.	France	Vandewalle-Capo et al., 2017
	1	BMD	92	Undefined	Clin.	England and Wales	Wilson et al., 2018 *
	1	E-test	183	Sg1	Clin.	Netherlands	Bruin et al., 2012
	0.5	E-test	100	Undefined	Env.	Southern Italy	De Giglio et al., 2015 *
	0.5	E-test	122	Undefined	Clin. + Env.	Norway	Natas et al., 2019 *
	1	E-test	149	Sg1	Clin. + Env.	China	Jia et al., 2019
	0.5	E-test	105	Undefined	Clin. + Env.	Northern Israel	Sharaby et al., 2019
	0.5	BMD	1464	undefined	Env.	China	This study
CLA	0.064	BMD	109	Sg1	Clin.	France	Vandewalle-Capo et al., 2017
	0.032	BMD	92	Undefined	Clin.	England and Wales	Wilson et al., 2018 *
	0.5	E-test	183	Sg1	Clin.	Netherlands	Bruin et al., 2012
	0.5	E-test	100	Undefined	Env.	Southern Italy	De Giglio et al., 2015 *
	0.5	E-test	122	Undefined	Clin. + Env.	Norway	Natas et al., 2019 *
	0.5	E-test	105	Undefined	Clin. + Env.	Northern Israel	Sharaby et al., 2019
	0.125	BMD	1464	Undefined	Env.	China	This study
AZI	2	BMD	109	Sg1	Clin.	France	Vandewalle-Capo et al., 2017
	0.25	BMD	50	Undefined	Clin. + Env.	England and Wales	Portal et al., 2021b *
	1	E-test	183	Sg1	Clin.	Netherlands	Bruin et al., 2012
	0.25	E-test	100	Undefined	Env.	Southern Italy	De Giglio et al., 2015 *
	0.25	E-test	122	Undefined	Clin. + Env.	Norway	Natas et al., 2019 *
	1	E-test	149	Sg1	Clin. + Env.	China	Jia et al., 2019
	2	E-test	105	Undefined	Clin. + Env.	Northern Israel	Sharaby et al., 2019
	0.5	BMD	1464	Undefined	Env.	China	This study
CIP	0.064	BMD	109	Sg1	Clin.	France	Vandewalle-Capo et al., 2017
	0.032	BMD	92	Undefined	Clin.	England and Wales	Wilson et al., 2018 *
	1	E-test	183	Sg1	Clin.	Netherlands	Bruin et al., 2012
	1	E-test	100	Undefined	Env.	Southern Italy	De Giglio et al., 2015 *
	1	E-test	122	Undefined	Clin. + Env.	Norway	Natas et al., 2019 *
	4	E-test	105	Undefined	Clin. + Env.	Northern Israel	Sharaby et al., 2019
	0.125	BMD	1464	Undefined	Env.	China	This study
MOX	0.064	BMD	109	Sg1	Clin.	France	Vandewalle-Capo et al., 2017
	0.125	BMD	92	Undefined	Clin.	England and Wales	Wilson et al., 2018 *
	1	E-test	183	Sg1	Clin.	Netherlands	Bruin et al., 2012
	1	E-test	100	Undefined	Env.	Southern Italy	De Giglio et al., 2015 *
	1	E-test	122	Undefined	Clin. + Env.	Norway	Natas et al., 2019 *
	1	E-test	149	Sg1	Clin. + Env.	China	Jia et al., 2019
	0.125	BMD	1464	Undefined	Env.	China	This study
LEV	0.032	BMD	109	Sg1	Clin.	France	Vandewalle-Capo et al., 2017
	0.125	BMD	92	Undefined	Clin.	England and Wales	Wilson et al., 2018 *
	0.125	BMD	50	Undefined	Clin.&Env.	England and Wales	Portal et al., 2021b *
	0.5	E-test	183	Sg1	Clin.	Netherlands	Bruin et al., 2012
	0.25	E-test	100	Undefined	Env.	Southern Italy	De Giglio et al., 2015 *
	0.25	E-test	122	Undefined	Clin. + Env.	Norway	Natas et al., 2019 *
	0.5	E-test	149	Sg1	Clin. + Env.	China	Jia et al., 2019
	2	E-test	105	Undefined	Clin. + Env.	Northern Israel	Sharaby et al., 2019
	0.063	BMD	1464	Undefined	Env.	China	This study
DOX	2	BMD	109	Sg1	Clin.	France	Vandewalle-Capo et al., 2017
	32	BMD	50	Undefined	Clin.&Env.	England and Wales	Portal et al., 2021b *
	8	E-test	183	Sg1	Clin.	Netherlands	Bruin et al., 2012
	8	E-test	100	Undefined	Env.	Southern Italy	De Giglio et al., 2015 *
	8	E-test	122	Undefined	Clin. + Env.	Norway	Natas et al., 2019 *
	0.5	E-test	105	Undefined	Clin. + Env.	Northern Israel	Sharaby et al., 2019
	32	BMD	1464	Undefined	Env.	China	This study

Env. indicates environmental sources, Clin. indicates clinical source. Cells filled with gray indicate similar results to those obtained by the present study (filled with light brown). *indicates that the ECOFFs were not directly shown in the original articles, and were based on the tentative highest MIC for wild-type organisms reported by the EUCAST—European Committee on Antimicrobial Susceptibility Testing—Guidance Document on Antimicrobial Susceptibility Testing of Legionella pneumophila. Available online: https://www.eucast.org/fileadmin/src/media/PDFs/EUCAST_files/Guidance_documents/Legionella_guidance_note_-_20210528.pdf.

### Detection of azithromycin resistance genes

**FIGURE 5:**
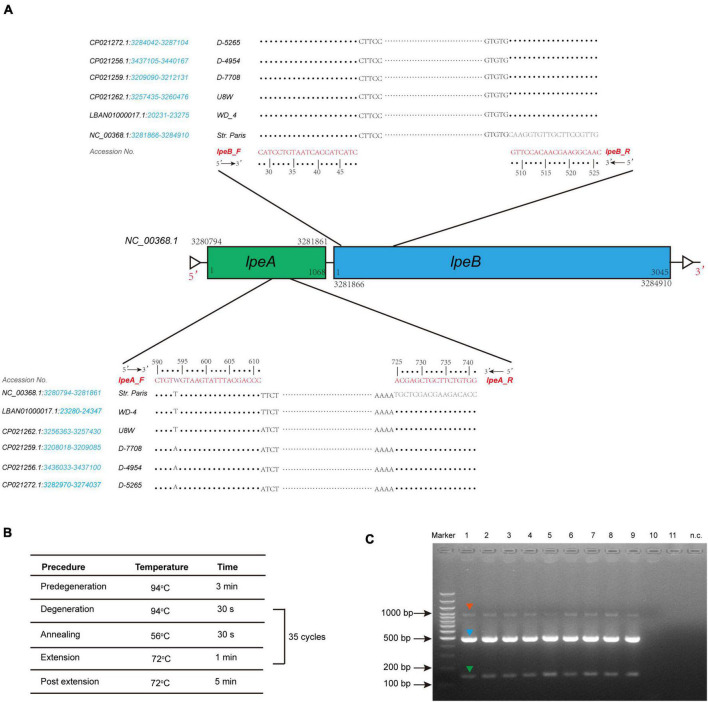
Design of *lepAB* primers with genomic sequences from *L. pneumophila* harboring the lpeAB efflux, and multiplex PCR to detect *lpeAB* genes and their spatial arrangement. **(A)** Arrangement of the *lpeAB* is shown using *L. pneumophila* str. Paris as a reference. Primers and DNA sequences of *lpeAB*, and GenBank accession numbers are shown, and the blue italics indicate the gene location in the selected sequences of the strains. **(B)** PCR amplification conditions. **(C)** Representative results for detection of *lpeAB* efflux in *L. pneumophila* isolates. DNA electrophoresis shows specific *lpeA* targets (green arrow, 152 bp), *lpeB* targets (blue arrow, 499 bp), and *lpeA-lpeB* combined targets (red arrow, 1009 bp), which indicate the right arrangement of the two genes. Lanes 1-9 show positive for *lpeAB* efflux; lanes 10-11 show negative for *lpeAB* efflux; n.c. indicates negative control which used sterile water as a template.

**FIGURE 6:**
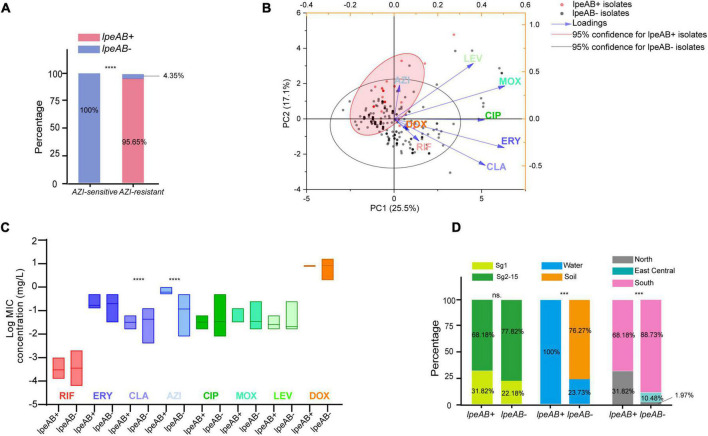
Presence of *lpeAB* efflux in *L. pneumophila* and its distribution in the isolates belonging to different sgs or from different sources and regions in China. **(A)** Presence of lpeAB efflux in AZI-sensitive and –resistant isolates. **(B)** PCA biplot of indices of the isolates harboring or not harboring *lpeAB* efflux. Isolates are shown as dots and colored by groups based on the presence of *lpeAB* efflux. Indices showed as lines with arrows and colored. The configuration of indices in the biplot represented the relationship between variables and principal components. The red shadow indicates 95% confidence for *lpeAB* + isolates. **(C)** Average MICs between isolates with or without *lpeAB* efflux. Data are shown as floating bars with the max and min and mean values. **(D).** Different distribution patterns of *lpeAB* in the isolates belonging to different sgs or from different sources and regions of China. *** *P* < 0.001, ****P < 0.0001.

To uncover possible resistance mechanisms associated with *L. pneumophila* against AZI, a multiplex PCR was conducted not only to detect the efflux pump coding genes *lpeA* and *lpeB* but also to detect the genetic arrangement of the two genes because the arrangement may have a potential impact on the gene expression (Ueda et al., 2005; Mernke et al., 2011). Our design differed from previous studies that only detected the presence of the two genes (Massip et al., 2017; Vandewalle-Capo et al., 2017; Natas et al., 2019). The design and multiplex PCR conditions, as well as the examples of PCR results, are shown in Figure 5. Based on the genomic study of 123 *L. pneumophila* strains that harbor *lepAB* efflux pump, the spatial arrangement of the two genes was resolved. *LpeB* is located downstream of *lpeA*, with an interval of four DNA bases (Figure 5A and Supplementary Table 1). All 46 AZI-resistant isolates and representative AZI-sensitive isolates that covered all types of MICs (0.008-0.25 mg/L) were tested. Among the 46 AZI-resistant isolates, 44 (95.65%) harbored *lpeAB* efflux; 13 AZI-resistant isolates that had an MIC of 1 mg/L all (100%) harbored *lpeAB* efflux. For the 33 AZI-resistant isolates that had an MIC of 0.5 mg/L, 31 (93.94%) harbored *lpeAB* efflux. All the *lepAB-*positive (*lpeAB* +) isolates had the right spatial arrangement of two genes. Conversely, none of the AZI-sensitive isolates (MIC < 0.5 mg/L) harbored the *lpeAB* efflux according to the PCR for 273 representative isolates (Figure 6A and Supplementary Table 2). A biplot *via* PCA indicated the configuration of MIC profiles of *L. pneumophila* isolates, as shown in Figure 6B. The second component (PC2) could roughly separate *lpeAB* + isolates from *lpeAB-* isolates, with AZI having 35.7% contribution for PC2, but only 2.5% for PC1. *LpeAB* + isolates were not observed to have an elevated MIC of the other seven antimicrobials, including the other two macrolides (ERY and CLA), but an elevated MIC of CLA in *lpeAB-* isolates was observed (Figure 6C), indicating that the *lpeAB* efflux pump*-*mediated resistance in the *L. pneumophila* environmental isolates were restricted to AZI, but not the other macrolides such as ERY and CLA. This partially contradicted Massip et al. report that Δ *lepAB* mutant could also lead to decreased MICs of other macrolides including ERY, CLA, and spiramycin against *L. pneumophila* strain Paris (Massip et al., 2017). We did not observe differences in the distribution of *lpeAB* efflux between the sg1 and sg2-15 isolates, but significantly different distribution patterns of *lpeAB* between water and soil isolates or among isolates from different regions were found (Figure 6D). Together, these results indicated that the presence of the *lepAB* efflux pump was the most important AZI but not the other seven antimicrobials resistance mechanism for *L. pneumophila*. The distribution patterns of AZI-resistant genes (*lpeAB*) were distinct between isolates from different environmental sources or regions in China. Natas et al. and Jia et al. both showed that all AZI-resistant *L. pneumophila* isolates harbored an *lpeAB* efflux pump; however, we did find 2 AZI-resistant isolates without *lpeAB*. These isolates also showed similar MICs of other two macrolides, as well as other types of antibiotics, when compared with those *lpeAB-* AZI-sensitive isolates (Supplementary Table 2), indicating some undefined AZI-specific resistance mechanisms such as special ribosomal mutations, and other AZI-specific efflux pumps in *L. pneumophila* might exist and deserve further study (Descours et al., 2017).

## Conclusion

A large number of *L. pneumophila* isolates may provide an unbiased antimicrobial susceptibility profile and ECOFFs for the clinically used antibiotics against LD. *L. pneumophila*, which is hidden in the environment, is responsible for LD. Limited data on antimicrobial susceptibility of *L. pneumophila* have been obtained in China over the past few decades. Our data will guide future studies of the antibiotic sensitivity and resistance mechanism of pathogenic *L. pneumophila*, which could serve as a reference for setting clinical breakpoints and discovering antibiotic-resistant *L. pneumophila* isolates in the clinic, as well as an effective treatment of *L. pneumophila* infection. Our data showed antimicrobial susceptibility of *L. pneumophila* belonging to different sgs or from different environmental sources and regions was distinct in some antimicrobial agents, such as RIF, ERY, CLA, and AZI. Most of the environmental isolates showed *in vitro* sensitivity to the tested antibiotics, in agreement with previously published data. In total, two isolates with fluoroquinolones (CIP, MOX, LEV) resistance and 46 isolates with AZI resistance were determined in the present study. Genomic research, multiplex PCR assay, and statistical analysis confirmed the correction of *lepAB* efflux with AZI-resistance in *L. pneumophila.* However, we found this mechanism was only restricted to AZI but no other macrolides (e.g., ERY and CLA). Furthermore, some undefined AZI-specific resistance mechanisms that are not the same as other macrolides (e.g., CLA and ERY) in *L. pneumophila* might exist. With the statistical analysis using our big data, we also proposed the existence of the shared defense strategies, particularly against both RIF and CLA in *L. pneumophila*, which deserves further research.

## Data availability statement

The original contributions presented in this study are included in the article/Supplementary material, further inquiries can be directed to the corresponding author.

## Author contributions

X-YZ designed the study, made data interpretation, and revised the manuscript. J-LY carried out the experiments. X-YZ and J-LY analyzed the data and wrote the manuscript. HS collected the environmental samples for the isolation of *Legionella* bacteria. J-LY, HS, XZ, and MY verified the data. All authors contributed to the article and approved the submitted version.
