# Corrigendum: Antimicrobial susceptibility profiles and tentative epidemiological cutoff values of *Legionella pneumophila* from environmental water and soil sources in China

**DOI:** 10.3389/fmicb.2022.1022197

**Published:** 2022-10-11

**Authors:** Jin-Lei Yang, Honghua Sun, Xuefu Zhou, Mo Yang, Xiao-Yong Zhan

**Affiliations:** The Seventh Affiliated Hospital, Sun Yat-sen University, Shenzhen, China

**Keywords:** *Legionella pneumophila*, antimicrobial susceptibility, epidemiological cut-off values, rifampin, clarithromycin, azithromycin, fluoroquinolones, *lpeAB*

In the published article, there was a mistake in Table 1 and Table 3. In Table 1, first, the subrow corresponding to “Sg2-15” is “Sg1” which is the abbreviation of Serogroup 1, not “Sg1 5” that is shown in the published article. Second, the columns of MIC_50_, MIC_90_, MIC range, and MIC diversities for the row RIF, subrow Sg2-15 should be as in this order: “0.0005, 0.0005, 0.0000625–0.002, and 0.57,” not the “0.0005, 0.000625–0.002, 0.57, and blank”. In Table 3, in the column “Regions of isolates” all the regions in the cells named “Southern Italy 477 Italy” should be “Southern Italy”. The corrected Tables 1, 3, and their captions appear below.

**Table 1:** Minimum inhibitory concentration (MIC) data of eight antimicrobials for the 1464 *L. pneumophila* isolates.

**Antibiotics**		**No. of** ***L. pneumophila*** **isolates inhibited at indicated concentrations (mg/L)**																							
		**0.0000625**	**0.000125**	**0.00025**	**0.0005**	**0.001**	**0.002**	**0.004**	**0.008**	**0.016**	**0.031**	**0.063**	**0.125**	**0.25**	**0.5**	**1**	**2**	**4**	**8**	**16**	**32**	**MIC**_**50**_	**MIC**_**90**_	**MIC range**	**MIC diversities**
RIF	All	18	77	580	749	37	3															0.0005	0.0005	0.0000625–0.002	0.58
	Sg1	1	20	183	119	5	1															0.00025	0.0005	0.0000625–0.002	0.56
	Sg2-15	17	57	397	630	32	2															0.0005	0.0005	0.0000625–0.002	0.57
ERY	All										3	117	552	494	298							0.25	0.5	0.031–0.5	0.70
	Sg1											63	182	74	10							0.125	0.25	0.063–0.5	0.61
	Sg2-15										3	54	370	420	288							0.25	0.5	0.031–0.5	0.69
CLA	All							1	3	46	748	661	5									0.031	0.063	0.004–0.125	0.53
	Sg1									13	233	82	1									0.031	0.063	0.008–0.125	0.44
	Sg2-15							1	3	33	515	579	4									0.063	0.063	0.004–0.125	0.53
AZI	All								1	0	2	264	1005	146	33	13						0.125	0.25	0.008–1	0.49
	Sg1											92	196	11	30							0.125	0.25	0.063–0.5	0.56
	Sg2-15								1	0	2	172	809	135	3	13						0.125	0.25	0.008–1	0.46
CIP	All								3	103	1160	195	1	0	2							0.031	0.063	0.008–0.5	0.35
	Sg1								1	12	276	37	1	0	2							0.031	0.063	0.008–0.5	0.28
	Sg2-15								2	91	884	158										0.031	0.063	0.008–0.063	0.37
MOX	All									20	1327	51	64	2								0.031	0.031	0.016–0.25	0.18
	Sg1									3	307	15	2	2								0.031	0.031	0.016–0.25	0.13
	Sg2-15									17	1020	36	62									0.031	0.031	0.016–0.125	0.19
LEV	All									965	451	46	0	2								0.016	0.031	0.016–0.25	0.47
	Sg1									182	140	5	0	2								0.016	0.031	0.016–0.25	0.51
	Sg2-15									783	311	1										0.016	0.031	0.016–0.063	0.41
DOX	All																1	58	1279	126		8	8	2–16	0.23
	Sg1																	19	285	25		8	8	4–16	0.24
	Sg2-15																1	39	994	101		8	8	2–16	0.22

The first column of the tables shows names of the antibiotics. The antibiotics belonging to the same class are filled with same color, shown as light red for rifampicin, light blue for macrolides, light green for fluoroquinolones, and light orange for tetracyclines. Other cells filled with colors indicate the concentration ranges of the antibiotics that were used for MIC determination.

**Table 3:** Epidemiological cutoff values (ECOFFs) of antimicrobials for *L. pneumophila* that are described in other articles.

**Antibiotics**	**ECOFFs** **(WT ≤ X mg/L)**	**Methods**	**Number of** **isolates**	**Sg of** **isolates**	**Sources**	**Regions of** **isolates**	**Ref**.
	0.001	BMD	109	Sg1	Clin.	France	Vandewalle-Capo et al., 2017
	0.008	BMD	50	Undefined	Clin.+Env.	England and Wales	Portal et al., 2021b *
RIF	0.008	BMD	92	Undefined	Clin.	England and Wales	Wilson et al., 2018*
	0.032	E-test	183	Sg1	Clin.	Netherlands	Bruin et al., 2012
	0.032	E-test	100	Undefined	Env.	Southern Italy	De Giglio et al., 2015*
	0.032	E-test	122	Undefined	Clin.+Env.	Norway	Natas et al., 2019*
	0.032	E-test	149	Sg1	Clin.+Env.	China	Jia et al., 2019
	0.063	E-test	105	Undefined	Clin.+Env.	Northern Israel	Sharaby et al., 2019
	0.002	BMD	1464	Undefined	Env.	China	This study
ERY	1	BMD	109	Sg1	Clin.	France	Vandewalle-Capo et al., 2017
	1	BMD	92	Undefined	Clin.	England and Wales	Wilson et al., 2018*
	1	E-test	183	Sg1	Clin.	Netherlands	Bruin et al., 2012
	0.5	E-test	100	Undefined	Env.	Southern Italy	De Giglio et al., 2015*
	0.5	E-test	122	Undefined	Clin.+Env.	Norway	Natas et al., 2019*
	1	E-test	149	Sg1	Clin.+Env.	China	Jia et al., 2019
	0.5	E-test	105	Undefined	Clin.+Env.	Northern Israel	Sharaby et al., 2019
	0.5	BMD	1464	undefined	Env.	China	This study
CLA	0.064	BMD	109	Sg1	Clin.	France	Vandewalle-Capo et al., 2017
	0.032	BMD	92	Undefined	Clin.	England and Wales	Wilson et al., 2018*
	0.5	E-test	183	Sg1	Clin.	Netherlands	Bruin et al., 2012
	0.5	E-test	100	Undefined	Env.	Southern Italy	De Giglio et al., 2015*
	0.5	E-test	122	Undefined	Clin.+Env.	Norway	Natas et al., 2019*
	0.5	E-test	105	Undefined	Clin.+Env.	Northern Israel	Sharaby et al., 2019
	0.125	BMD	1464	Undefined	Env.	China	This study
AZI	2	BMD	109	Sg1	Clin.	France	Vandewalle-Capo et al., 2017
	0.25	BMD	50	Undefined	Clin.+Env.	England and Wales	Portal et al., 2021b*
	1	E-test	183	Sg1	Clin.	Netherlands	Bruin et al., 2012
	0.25	E-test	100	Undefined	Env.	Southern Italy	De Giglio et al., 2015*
	0.25	E-test	122	Undefined	Clin.+Env.	Norway	Natas et al., 2019*
	1	E-test	149	Sg1	Clin.+Env.	China	Jia et al., 2019
	2	E-test	105	Undefined	Clin.+Env.	Northern Israel	Sharaby et al., 2019
	0.5	BMD	1464	Undefined	Env.	China	This study
CIP	0.064	BMD	109	Sg1	Clin.	France	Vandewalle-Capo et al., 2017
	0.032	BMD	92	Undefined	Clin.	England and Wales	Wilson et al., 2018*
	1	E-test	183	Sg1	Clin.	Netherlands	Bruin et al., 2012
	1	E-test	100	Undefined	Env.	Southern Italy	De Giglio et al., 2015*
	1	E-test	122	Undefined	Clin.+Env.	Norway	Natas et al., 2019*
	4	E-test	105	Undefined	Clin.+Env.	Northern Israel	Sharaby et al., 2019
	0.125	BMD	1464	Undefined	Env.	China	This study
MOX	0.064	BMD	109	Sg1	Clin.	France	Vandewalle-Capo et al., 2017
	0.125	BMD	92	Undefined	Clin.	England and Wales	Wilson et al., 2018*
	1	E-test	183	Sg1	Clin.	Netherlands	Bruin et al., 2012
	1	E-test	100	Undefined	Env.	Southern Italy	De Giglio et al., 2015*
	1	E-test	122	Undefined	Clin.+Env.	Norway	Natas et al., 2019*
	1	E-test	149	Sg1	Clin.+Env.	China	Jia et al., 2019
	0.125	BMD	1464	Undefined	Env.	China	This study
LEV	0.032	BMD	109	Sg1	Clin.	France	Vandewalle-Capo et al., 2017
	0.125	BMD	92	Undefined	Clin.	England and Wales	Wilson et al., 2018*
	0.125	BMD	50	Undefined	Clin.&Env.	England and Wales	Portal et al., 2021b*
	0.5	E-test	183	Sg1	Clin.	Netherlands	Bruin et al., 2012
	0.25	E-test	100	Undefined	Env.	Southern Italy	De Giglio et al., 2015*
	0.25	E-test	122	Undefined	Clin.+Env.	Norway	Natas et al., 2019*
	0.5	E-test	149	Sg1	Clin.+Env.	China	Jia et al., 2019
	2	E-test	105	Undefined	Clin.+Env.	Northern Israel	Sharaby et al., 2019
	0.063	BMD	1464	Undefined	Env.	China	This study
DOX	2	BMD	109	Sg1	Clin.	France	Vandewalle-Capo et al., 2017
	32	BMD	50	Undefined	Clin.&Env.	England and Wales	Portal et al., 2021b*
	8	E-test	183	Sg1	Clin.	Netherlands	Bruin et al., 2012
	8	E-test	100	Undefined	Env.	Southern Italy	De Giglio et al., 2015*
	8	E-test	122	Undefined	Clin.+Env.	Norway	Natas et al., 2019*
	0.5	E-test	105	Undefined	Clin.+Env.	Northern Israel	Sharaby et al., 2019
	32	BMD	1464	Undefined	Env.	China	This study

Env. indicates environmental sources, Clin. indicates clinical source. Cells filled with gray indicate similar results to those obtained by the present study (filled with light brown).

*indicates that the ECOFFs were not directly shown in the original articles, and were based on the tentative highest MIC for wild-type organisms reported by the EUCAST—European Committee on Antimicrobial Susceptibility Testing—Guidance Document on Antimicrobial Susceptibility Testing of Legionella pneumophila. Available online: https://www.eucast.org/fileadmin/src/media/PDFs/EUCAST_files/Guidance_documents/Legionella_guidance_note_-_20210528.pdf.

The authors apologize for this error and state that this does not change the scientific conclusions of the article in any way. The original article has been updated.

## Publisher's note

All claims expressed in this article are solely those of the authors and do not necessarily represent those of their affiliated organizations, or those of the publisher, the editors and the reviewers. Any product that may be evaluated in this article, or claim that may be made by its manufacturer, is not guaranteed or endorsed by the publisher.

