# Supplementary material for: Antimicrobial susceptibility profiles and tentative epidemiological cutoff values of Legionella pneumophila from environmental water and soil sources in China
Source: Front Microbiol. 2022 Aug 18;13:924709. doi: 10.3389/fmicb.2022.924709 (PMC9597688; doi:10.3389/fmicb.2022.924709)
Supplement: Supplementary file 3 [file Data_Sheet_1.PDF]

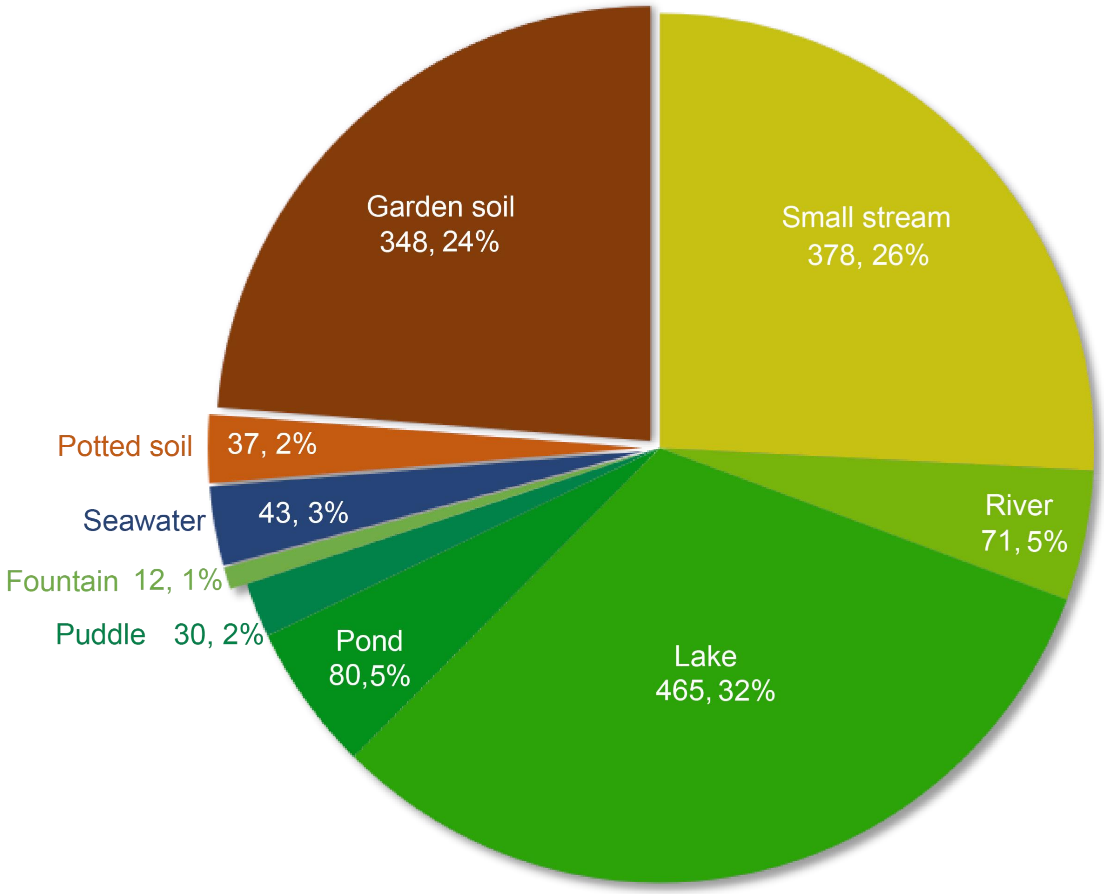

Supplementary Figure 1. The pie chart shows the numbers and their percentages of *L. pneumophila* isolates from various environmental sources of China that were used for the antimicrobial susceptibility tests.
