# Supplementary material for: Antimicrobial susceptibility profiles and tentative epidemiological cutoff values of Legionella pneumophila from environmental water and soil sources in China
Source: Front Microbiol. 2022 Aug 18;13:924709. doi: 10.3389/fmicb.2022.924709 (PMC9597688; doi:10.3389/fmicb.2022.924709)
Supplement: Supplementary file 4 [file Data_Sheet_2.PDF]

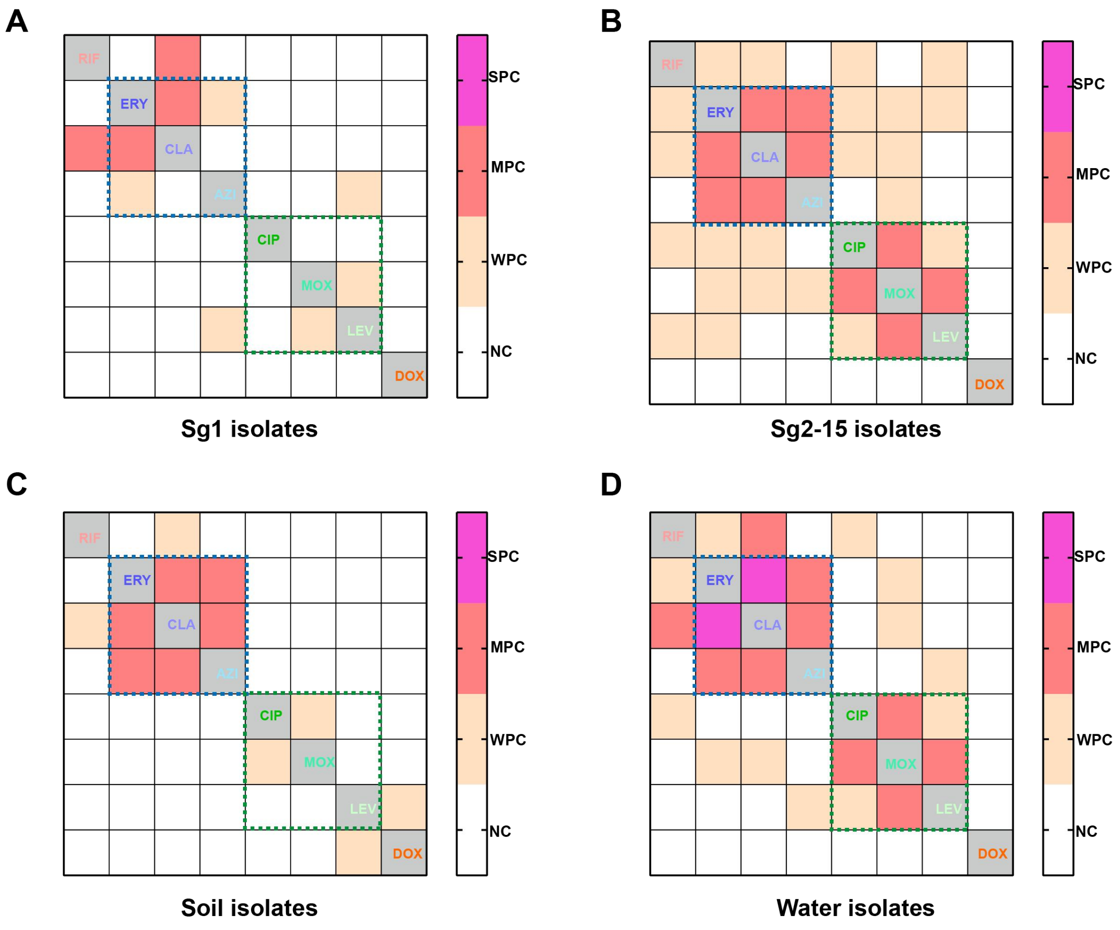

Supplementary Figure 2. Correlation matrices for the MICs were obtained from the 329 sg1 *L. pneumophila* isolates. (A), or 1135 sg2-15 isolates (B), 385 soil isolates (C), or 1079 water isolates (D). WPC, weak positive correlation, 0.2<r≤0.3; MPC, moderate positive correlation, 0.3<r≤0.5; SPC, strong positive correlation, r>0.5. NC, no correlation. The dotted lines indicate that these antibiotics belong to the same class.
